# Factors Associated With Mortality Among Adult Hemorrhagic Stroke Patients in Public Hospitals in the Harari Region, Eastern Ethiopia: A Retrospective Hospital‐Based Cohort Study

**DOI:** 10.1155/srat/9554811

**Published:** 2026-08-20

**Authors:** Alemayehu Tesfaye, Birhanu Shegene, Abdi Gari Negasa, Akewok Sime, Obsan Kassa, Dawit Firdisa

**Affiliations:** ^1^ School of Public Health, College of Health and Medical Sciences, Haramaya University, Harar, Ethiopia, haramaya.edu.et; ^2^ School of Medical Laboratory Science, College of Health and Medical Sciences, Haramaya University, Harar, Ethiopia, haramaya.edu.et

**Keywords:** eastern Ethiopia, factors, hemorrhagic stroke patients, mortality

## Abstract

**Background:**

A hemorrhagic stroke is a life‐threatening emergency that can lead to disability or death without prompt treatment. However, there is limited evidence on factors associated with mortality in hemorrhagic stroke, particularly in eastern Ethiopia. Therefore, this study is aimed at assessing the factors associated with mortality among adult hemorrhagic stroke patients in the Harari region, eastern Ethiopia.

**Methodology:**

A retrospective cohort study was conducted among 262 patients with hemorrhagic stroke at public hospitals in the Harari region of eastern Ethiopia, from September 1, 2019, to August 31, 2024. Factors associated with mortality were identified using the Gompertz hazards regression model.

**Results:**

Of the 262 patients included in the final analysis, 45 (17.18%; 95% CI: 13.05%–22.26%) died. The mortality rate for hemorrhagic stroke was 10.62 cases per 1000 person‐months (95% CI: 7.93–14.23). Mortality was significantly associated with patients having kidney disease (AHR: 3.04; 95% CI: 1.44–6.44; *p* value: 0.004) and poor Glasgow Coma Scale (GCS) scores (AHR: 3.92; 95% CI: 1.58–9.71; *p* value: 0.003). Septic shock and aspiration pneumonia had exploratory associations with mortality.

**Conclusion:**

In‐hospital mortality was observed in approximately one in six hemorrhagic stroke patients treated at public hospitals. Risk factors such as kidney disease, aspiration pneumonia, poor GCS, and septic shock increased mortality risk. Hence, special emphasis should be given to early screening for kidney disease and the management of stroke patients with poor a GCS score.

## 1. Introduction

Hemorrhagic stroke is a life‐threatening neurological emergency that requires immediate medical intervention, as delayed diagnosis and treatment are associated with an increased risk of severe neurological disability and mortality [1]. It occurs due to bleeding into the brain caused by the rupture of a blood vessel and is further subdivided into intracerebral hemorrhage (ICH) and subarachnoid hemorrhage [2, 3]. Acute lobar cerebral hemorrhages present with a different clinical presentation and a more severe early prognosis than deep subcortical ICHs. In addition, nonhypertensive mechanisms are more likely to predominate in the lobar location [4]. Compared with ischemic strokes, hemorrhagic strokes tend to present more severely with higher fatality, requiring different prevention and treatment strategies [5].

Although hemorrhagic stroke accounts for a smaller proportion of stroke cases than ischemic stroke, it contributes disproportionately to stroke‐related mortality and disability worldwide [6]. There were 62.8 million disability adjusted life years (DALYs) lost (86% in low‐ and middle‐income countries [LMICs]) due to hemorrhagic stroke [7]. Globally, ICH represents approximately 28% of all strokes but is associated with the highest mortality, with a case‐fatality rate of 35.5% and minimal improvement in survival over recent decades [6]. Beyond mortality, stroke survivors often face severe physical impairments such as paralysis, loss of speech, and cognitive difficulties, resulting in significant care burdens for families and health systems [8]. The impact is especially profound in LMIC, where healthcare resources for prevention, treatment, and rehabilitation are limited [9]. A recent study reported that hemorrhagic stroke patients in Africa had a pooled mortality rate of 26.1% (95% confidence interval [CI]: 24.0%–28.3%), notably higher than for ischemic stroke [10]. Stroke accounts for 6.23% of deaths in Ethiopia, with an age‐adjusted stroke mortality rate of 89.82 per 100,000 population [11]. A systematic review and meta‐analysis in Ethiopia showed that the percentage of hemorrhagic stroke cases among total stroke patients in Ethiopia is approximately 36.53% [12]. Many studies in Ethiopia have investigated stroke in general rather than focusing on particular types of strokes. Current research often pools both hemorrhagic and ischemic stroke patients or relies heavily on ischemic stroke data because ischemic stroke is the most common type and may dominate it [13, 14]. To the best of our knowledge, the burden of mortality among patients with hemorrhagic stroke has not been comprehensively characterized in Ethiopia. Identifying stroke subtypes is essential for guiding appropriate stroke management [15].

Understanding the factors associated with mortality in hemorrhagic stroke is crucial for improving clinical decision‐making, optimizing resource allocation, and guiding patient and family counseling. Factors such as patient demographics, pre‐existing comorbidities such as hypertension and diabetes, clinical presentation at admission, such as Glasgow Coma Scale (GCS) score, and the development of in‐hospital complications such as aspiration pneumonia and septic shock are known to influence survival [12, 16–19]. In addition, intraventricular extension of ICH and advanced age were determinants of poor outcome [20].

However, the specific risk factors and their magnitude can vary significantly across different populations and healthcare systems. It is important to study hemorrhagic stroke‐specific prognostic factors—such as hematoma volume, intraventricular extension, and initial GCS score—to enable accurate early risk stratification, improve the quality of patient and family counseling, and inform the development of targeted therapies designed to mitigate the high lethality of hemorrhagic events specifically. Given the current data gaps and the severity of hemorrhagic strokes, it is important to separately assess factors associated with mortality among hemorrhagic stroke patients. Therefore, this study is aimed at identifying the factors associated with mortality among adult hemorrhagic stroke patients admitted to public hospitals in the Harari region, eastern Ethiopia, using a retrospective hospital‐based cohort design. The findings will provide valuable, context‐specific evidence to clinicians and health policymakers, enabling them to improve the quality of care and survival outcomes for this critically ill patient group.

## 2. Methods

### 2.1. Study Design and Setting

A hospital‐based retrospective cohort study was conducted among patients diagnosed with hemorrhagic stroke at public hospitals in the Harari region, eastern Ethiopia, from September 1, 2019, to August 31, 2024. The Harari region is situated 526 km east of Addis Ababa, the capital city of Ethiopia. The region is currently served by two public hospitals. The two public hospitals in the area—Jugal General Hospital (JGH) and Hiwot Fana Comprehensive Specialized University Hospital (HFCSUH)—deliver medical services to the local population. The data were extracted from September 1 to October 1, 2024.

### 2.2. Population and Sampling

The source population for this study included all patients aged 15 years and older who were treated for hemorrhagic stroke at public hospitals in the Harari Regional State. The study population specifically consisted of hemorrhagic stroke patients who were admitted and registered in the adult medical wards of these hospitals during the study period. Patients with incomplete medical information on the treatment outcome of hemorrhagic stroke were excluded.

**Figure 1:**
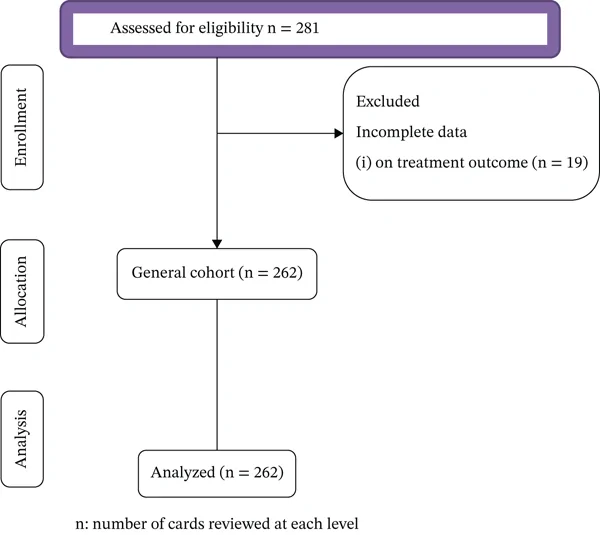
Flow diagram of selection for study on the factors associated with mortality among adult hemorrhagic stroke patients in the Harari region, east Ethiopia, 2024.

A total of 281 hemorrhagic stroke patients were treated at the medical ward of HFCSUH and JGH in the study period. There were 256 and 25 patients from each hospital, respectively. Of these, 19 patients′ medical records were incomplete. The final analysis included 262 hemorrhagic strokes. (Figure 1).

### 2.3. Variables

The primary outcome of this study was in‐hospital mortality among hemorrhagic stroke patients. Diagnosis of hemorrhagic stroke was based on clinical assessment in conjunction with neuroimaging. A noncontrast computed tomography (CT) scan was utilized to confirm the diagnosis. Data on mortality were obtained from the death summaries documented in patients′ medical records.

### 2.4. Operational Definitions

**Event**: the mortality of hemorrhagic stroke.

**Censored**: hemorrhagic stroke patients who did not die during the follow‐up study (improved, discharged against medical advice [DAMA], and discharged with complications).

**Treatment outcomes**: the results or effects of medical intervention on a patient′s health (improved, complications, or death) [21].

**Improved**: Information about the improvement of patients was obtained from the discharge summary of medical records. **Functional outcome** was evaluated by the modified Rankin scale (mRS). It was categorized into good (mRS < 3) and poor (mRS ≥ 3) functional recovery [22].

**A good GCS** indicates a patient with mild brain injury or who is alert (GCS 13–15), a moderate brain injury or who is drowsy (GCS 9–12), and a **poor GCS** indicates a patient with severe brain injury or who is unconscious (GCS 3–8) [23].

**Time to death**: time from the date of hemorrhagic stroke diagnosis to the date of death [24].

### 2.5. Data Collection and Quality Control

Data were collected using a data extraction form adapted from the WHO STEPSwise approach to stroke surveillance [25]. This form included key patient information, including demographic details (age, sex, and residency), clinical data (hypertension, atrial fibrillation, DM, aspiration pneumonia, kidney disease, and raised intracranial pressure [ICP]), treatment outcomes, medications used, and laboratory investigations. Four public health professionals from the university conducted data collection under the supervision of two trained supervisors. Data were extracted from patients′ paper‐based medical records.

Before the actual data collection, a pretest was conducted using 5% (14) of the medical records of stroke patients from both hospitals to assess the reliability and consistency of the data collection tools. Based on the pretest findings, necessary revisions were made to the instruments, and the data were not included in the final analytical sample. Data collectors received training for 2 days on the procedures and tools of data collection. Data collection was closely supervised by the principal investigators, and any inconsistencies identified were promptly addressed. Data collected using the Kobo toolbox were reviewed for consistency and completeness before being uploaded to the server.

### 2.6. Data Analysis

**Figure 2:**
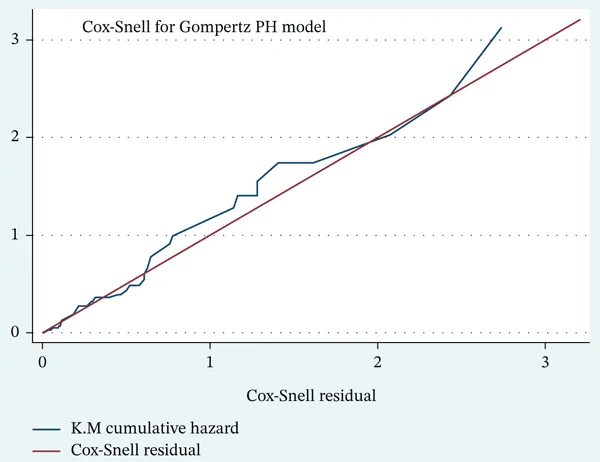
Cox–Snell residuals for Gompertz PH models of hemorrhagic stroke patients in the public hospitals in the Harari region, eastern Ethiopia, 2024.

The dataset was then imported into STATA Version 17.0 for analysis. Descriptive statistics, including means, frequencies, tables, and graphs, were used to summarize and describe the data. To address missing data, a complete case analysis was applied under the assumption that data were missing completely at random (MCAR). The cumulative incidence of mortality was calculated by dividing the number of deaths by the total initial population at risk during follow‐up. Patient‐months at risk were determined from the date of baseline diagnosis to either the date of the event (death) or censoring. Based on this, incidence density was calculated as the number of deaths per patient‐months at risk. The outcome variable was categorized into two groups: death (event) and censored. Kaplan–Meier failure curves were used to estimate mortality probabilities, and the log‐rank test was employed to compare survival functions across explanatory variables. To identify factors associated with mortality, both the Cox proportional hazards (PH) model and three parametric survival models (Weibull, Exponential, and Gompertz) were fitted. The model was selected by the Akaike information criterion (AIC), Bayesian information criterion (BIC), and log‐likelihood criterion, with the Gompertz PH model ultimately chosen as the most parsimonious. In addition, the selection was guided by the suitability of the Gompertz distribution for modeling monotonically changing hazards, which is clinically plausible in hemorrhagic stroke, where the risk of mortality may evolve consistently over time. The direction and magnitude of the effect estimates were broadly consistent across models. The PH assumption was checked using Schoenfeld residuals (both global and scaled) as well as graphical methods, such as the log–log survival plots. Multicollinearity among factors was assessed using the variance inflation factor (VIF). The following variables were selected on clinical and theoretical grounds: age greater than 65 years, atrial fibrillation, kidney diseases, brain edema, and GCS [24, 26–30]. A *p* value < 0.05 was considered statistically significant in the multivariable model, and the strength of associations was expressed using hazard ratios (HR) with 95% CIs. The goodness of fit of the model was assessed via the Cox–Snell residual technique. The cumulative hazard plot follows a straight line through the origin with slope, indicating that the model fits the data well. (Figure 2).

## 3. Results

### 3.1. Sociodemographic Characteristics

Out of 281 adult hemorrhagic stroke patient records reviewed, 262 were included in the final analysis. A total of 19 records (6.76%) were excluded due to incomplete information. Among the 262 adults diagnosed with hemorrhagic stroke, 171 (65.27%) were male, with a mean age of 55 years (±15.67 SD). Regarding residence, approximately 65.27% of patients were from rural areas (Table 1).

**Table 1:** Sociodemographic characteristics of hemorrhagic stroke patients at public hospitals in the Harari region, east Ethiopia, from 2019 to 2024 (*N* = 262).

Characteristics	Category	Frequency (%)
Sex	Male	171 (65.27)
	Female	91 (34.73)
Age	< 45	70 (26.72)
	45–65	125 (47.71)
	> 65	67 (25.57)
Residence	Rural	170 (64.89)
	Urban	(35.11)

### 3.2. Clinical Characteristics

The most frequently observed clinical presentation was hemiparesis (72.90%), followed by loss of consciousness (37.79%). The median time from symptom onset to hospital presentation was 29 h (IQR: 60), and in about 50.38% of cases, the time from symptom onset to admission exceeded 24 h. A large proportion of patients, 195 (74.43%), had at least one comorbid condition. Hypertension was the most common comorbidity (60.31%), followed by diabetes (11.45%) and kidney disease (9.54%). At the time of hospitalization, the mean diastolic and systolic blood pressures were 83.85 ± 19.73 and 140.98 ± 28.55 mmHg, respectively. Elevated body temperature (> 37.5°C) was observed in 9.54% of the patients. The mean total cholesterol level was 149.43 ± 50.81 mg/dL, with 17.57% of patients showing elevated levels. The median length of hospital stay was 5 days (±6 SD) (Table 2).

**Table 2:** Clinical characteristics of hemorrhagic stroke patients at public hospitals in the Harari region, east Ethiopia, from 2019 to 2024 (*N* = 262).

Characteristics	Category	Frequency (%)
Clinical presentations	Hemiparesis	191 (72.90)
	Loss of consciousness	99 (37.79)
	Slurred speech	63 (24.05)
	Headache	69 (26.34)
	Aphasia	40 (15.27)
	Vomiting	47 (17.94)
	Facial palsy	35 (13.36)
Previous history of stroke	No	219 (83.91)
	Yes	42 (16.09)
Time from onset to admission (in hours)	Median ± (IQR) = 29 ± 60	
Comorbidity	Yes	195 (74.43)
	No	67 (25.57)
List of comorbidities	Hypertension	158 (60.31)
	Heart failure	20 (7.63)
	Diabetes	30 (11.45)
	Kidney disease	25 (9.54)
	Myocardial Infraction	6 (2.29)
	Atrial fibrillation	11 (4.20)
Systolic blood pressure (in mmHg)	Mean ± (SD) = 140.98 ± 28.55	
Diastolic blood pressure (in mmHg)	Mean ± (SD) = 83.85 ± 19.73	
Respiratory rate (in breaths per minute)	12–18	16 (6.11)
	> 18	246 (93.89)
Pulse rate (in beats per minute)	< 60	8 (3.05)
	60–100	189 (72.14)
	> 100	65 (24.81)
Body temperature (in °C)	< 36.5	109 (41.60)
	36.5–37.5	128 (48.85)
	> 37.5	25 (9.54)
Random blood glucose (in mg/dL)	≤ 200	248 (94.66)
	> 200	14 (5.34)
Total cholesterol (mg/dL)	< 200	183 (82.43)
	≥ 200	39 (17.57)
Serum creatinine (in mg/dL)	< 0.5	21 (8.08)
	0.5–1.2	196 (75.38)
	> 1.2	43 (16.53)
Serum potassium level (in meq/L)	< 3.5	58 (22.39)
	3.5–5	190 (73.36)
	> 5	11 (4.25)
Glasgow Coma Scale (GCS)	≤ 8	43 (16.41)
	9–12	95 (36.26)
	13–15	124 (47.33)
Length of hospital stays(days)	Median ± (IQR) = 5 ± 6	

### 3.3. Treatment Characteristics

Among the 262 patients, 174 (66.41%; 95% CI: 60.43%–71.91%) had improved outcomes, 10 (3.82%; 95% CI: 2.05%–6.97%) were discharged with complications, 45 (17.18%; 95% CI: 13.05%–22.26%) died, and 33 (12.60%; 95% CI: 9.07%–17.22%) were discharged against medical advice on self and family requests. Among the 45 patients who died, aspiration pneumonia (57.78%) and increased ICP (55.56%) were identified as frequently documented contributing causes of death secondary to hemorrhagic stroke in the investigated patients, whereas hypertension (75.56%) was primarily an underlying risk factor that predisposes to the stroke. Of 262 patients, 129 (49.24%) experienced complications. The most common complications were increased ICP/brain edema (24.43%) and aspiration pneumonia (21.76%). Amlodipine (32.06%) and enalapril (29.77%) were frequently used antihypertensive medications. Additionally, antibiotics such as ceftriaxone and metronidazole were prescribed in 31.68% of patients, primarily for the treatment of aspiration pneumonia and sepsis associated with comorbid conditions. (Table 3)

**Table 3:** Treatment outcomes for hemorrhagic stroke patients at public hospitals in the Harari region, east Ethiopia, from 2019 to 2024 (*N* = 262).

Characteristics	Category	Frequency (%)
Treatment outcomes	Improved	174 (66.41)
	Discharged with complications	10 (3.82)
	Died	45 (17.18)
	DAMA	33 (12.60)
Complication	Yes	129 (49.24)
	No	133 (50.76)
Lists of complications	Brain edema/ICP	64 (24.43)
	Aspiration pneumonia	57 (21.76)
	UTI	16 (6.11)
	HAI	20 (7.63)
	Seizure	10 (3.82)
	Septic shock	13 (4.96)
Antihypertensive	Enalapril	78 (29.77)
	Amlodipine	84 (32.06)
	Nifedipine	38 (14.50)
	Hydrochlorothiazide	31 (11.83)
Antiulcer drugs	Cimetidine	43 (16.41)
	Omeprazole	13 (4.45)
Antibiotics	Ceftriaxone	77 (29.39)
	Metronidazole	6 (2.29)
Antipyretic	Paracetamol	79 (30.15)
Antidiabetic	Insulin	14 (5.34)
Osmotic diuretics	Mannitol	63 (24.05)

Abbreviations: DAMA, discharged against medical advice; HAI, hospital‐acquired infection; ICP, intracranial pressure; UTI, urinary tract infection.

### 3.4. Incidence Rate of Stroke Patients

**Figure 3:**
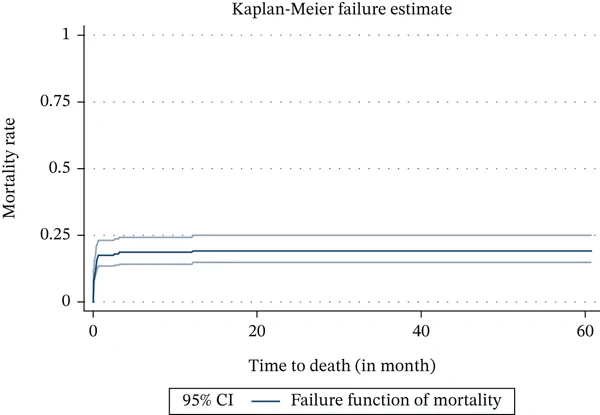
Overall Kaplan–Meier curve for the cumulative probability of mortality of hemorrhagic stroke patients at public hospitals in the Harari region, east Ethiopia, 2024.

The study samples were followed for a minimum of 1 day and a maximum of 60 months, with no median survival time. Among the total cohort of stroke patients, 45 (17.18%; 95% CI: 13.05%–22.26%) died during the follow‐up period. The overall incidence of death was 10.62 cases per 1000 person‐months of observation (95% CI: 7.93–14.23). The survival probability was highest in the first month of follow‐up after hemorrhagic stroke was diagnosed and then decreased as the follow‐up time increased (Figure 3).

### 3.5. Factors Associated With Mortality

In the multivariable Gompertz regression analysis, two variables—kidney disease and poor GCS scores—were identified as factors associated with mortality. Septic shock and aspiration pneumonia showed exploratory associations with mortality.

Accordingly, patients with kidney disease had a three fold greater hazard of death from hemorrhagic stroke than those without kidney disease (AHR: 3.04; 95% CI: 1.44, 6.44). The hazard of mortality from hemorrhagic stroke was 3.92 times greater among patients with a GCS score < 8 than among those with a GCS score of 13–15 (AHR: 3.92; 95% CI: 1.58, 9.71) (Table 4).

**Table 4:** Bivariate and multivariate Gompertz regression analyses of predictors of mortality among hemorrhagic stroke patients at public hospitals in the Harari region, east Ethiopia, 2024.

Variable	Category	Mortality		CHR (95% CI)	AHR (95% CI)	*p*
		Censored (*n* = 217)	Event (*n* = 45)			
Age	< 45	61	9	1	1	
	45–65	111	14	0.89 (0.38–2.05)	0.67 (0.27–1.65)	0.385
	> 65	45	22	2.57 (1.18–5.57)	0.78 (0.31–1.95)	0.598
Atrial fibrillation	No	211	40	1	1	
	Yes	6	5	2.69 (1.06–6.83)	1.26 (0.44–3.60)	0.657
Kidney diseases	No	208	29	1	1	
	Yes	9	16	6.91 (3.73–12.79)	3.04 (1.44–6.44)	**0.004**
Brain edema	No	178	20	1	1	
	Yes	39	25	4.77 (2.64–8.61)	1.58 (0.72–3.46)	0.253
GCS	< 8	23	19	14.31 (6.30–32.48)	3.92 (1.58–9.71)	**0.003**
	9–12	78	17	2.97 (1.27–6.95)	1.22 (0.48–3.12)	0.671
	13–15	159	6	1	1	

*Note:* Bold values denote significance at *p* < 0.05.

Similarly, the risk of mortality from hemorrhagic stroke was greater among patients with septic shock than among those without septic shock. Hemorrhagic stroke patients diagnosed with aspiration pneumonia were more likely to die than those without the condition.

## 4. Discussion

This study included 262 patients with hemorrhagic stroke; among the total number of hemorrhagic stroke patients followed for approximately 60 months, 45 (17.18%; 95% CI: 13.05%–22.26%) died. The incidence of mortality was 10.62 cases per 1000 person‐months of observation, with a 95% CI of 7.93–14.23. In the multivariable Gompertz regression analysis, two variables—kidney disease and poor GCS scores—were identified as factors associated with mortality. Additionally, septic shock and aspiration pneumonia showed exploratory associations with mortality.

The cumulative incidence of mortality observed in this study was consistent with the incidence of mortality of 19.38% reported in Debre Markos Comprehensive Specialized Hospital, Ethiopia [31], 20.64% in Jimma University Medical Center Stroke Unit, Ethiopia [32], 19.10% in Felege Hiwot Hospital, Ethiopia [24], 16.67% in Beirut [33], and 20.40% in Taiwan [34]. However, it was higher than the study done by [35] in the Aseer Region, southwest of Saudi Arabia, 9.7%. On the other hand, the cumulative incidence in this study was lower than the study conducted in Sierra Leone (33.9%) [36], at University of Gondar Teaching Hospital, Tibebe Gion Comprehensive Specialized Hospital, and Felege Hiwot Referral Hospital, Ethiopia (40.91%) [37], at Mettu Karl Referral, Ethiopia (41.43%) [38], at Jimma University Medical Center, Ethiopia (74.87%) [26], and in Tanzania (44.26%) [39]. This variation may be due to differences in socioeconomic status, level of awareness of the population on risk factors of stroke and its mortality, and differences in sample size and follow‐up time. In developing countries such as Ethiopia, deteriorating patients remaining at home against medical advice resulted in out‐of‐hospital mortality and may have contributed to an underestimated mortality rate of stroke patients [31].

Aspiration pneumonia showed an association with mortality among stroke patients. These results are consistent with studies done in Lusaka, Zambia [40], in Sierra Leone [36], in Tanzania [41], at Ayder Comprehensive Specialized Hospital, Mekelle, Ethiopia [28], and at Jimma University Medical Center, Ethiopia [27]. Aspirational pneumonia can lead to several respiratory problems, including acute respiratory distress syndrome and respiratory failure, which can worsen the course of treatment for hemorrhagic stroke patients and raise their chance of dying [42].

Likewise, the level of GCS < 8 score at the time of hospital admission was associated with hemorrhagic stroke patient mortality. This finding was in line with studies conducted in Saudi Arabia [35], Iran [43], South London [44], Ayder Comprehensive Specialized Hospital, Mekelle, Ethiopia [28], Lusaka, Zambia [40], Jimma University Medical Center, Ethiopia [27], and Saint Paul′s Hospital Millennium Medical College, Ethiopia [29]. The possible explanation could be that patients with decreased GCS or comatose patients had a higher probability of developing acute phase neuromedical stroke complications, which possibly predispose them to a high risk of death [31].

Having kidney disease was significantly associated with mortality among stroke patients, with affected patients demonstrating a 3.04‐fold higher hazard of death from hemorrhagic stroke than those without kidney disease (AHR: 3.04; 95% CI: 1.44, 6.44). This finding was in line with studies conducted in South Ethiopia [30], in India [45], and a review study [46]. Hemorrhagic stroke is the leading cause of death in the chronic kidney disease (CKD) population. Uremic patients are susceptible to hemorrhagic complications due to several factors, namely platelet dysfunction, low platelet number, use of heparin during hemodialysis, and use of anticoagulants for thromboembolic risk [47, 48].

Septic shock showed an association with mortality among stroke patients. This finding was in line with studies conducted in the California State Inpatient Database of the Healthcare Cost and Utilization Project data set [49]. The risk of stroke is high after sepsis, and this risk persists for up to a year. Younger sepsis patients have a particularly increased risk of stroke after sepsis [50].

A potential line for future research in hemorrhagic stroke could be the prediction and prevention of hematoma expansion and rebleeding in ICH through the development and validation of multimodal, precision risk models that combine dynamic imaging biomarkers, serial clinical data, and coagulation biomarkers. This is important because hematoma expansion (≥ 30 mL) is associated with poor ICH outcome [1, 51, 52]. Accordingly, prospective multicenter studies should evaluate whether model‐guided treatment reduces hematoma growth and improves outcomes.

## 5. The Limitations of the Study

Due to the retrospective design, incomplete or missing data were encountered, including instances of lost patient medical information. Consequently, several clinically important variables that may have a significant association with hemorrhagic stroke mortality could not be ascertained from the medical records and were not assessed. This limitation may have led to an underestimation of the observed effects. The absence of important clinical and neuroimaging severity indicators (e.g., hematoma volume, location, IVH, and stroke severity scores) may have led to incomplete adjustment for baseline risk. As a result, the observed effect size estimates may be biased. Moreover, because the study was hospital‐based and assumed hemorrhagic stroke to be the exclusive cause of all mortality events, the stroke‐specific mortality rate may have been overestimated.

## 6. Conclusion

In this study, the in‐hospital mortality rate among patients with hemorrhagic stroke was high. Risk factors such as kidney disease, aspiration pneumonia, poor GCS, and septic shock increased mortality risk. We recommend that priority be given to the management of hemorrhagic stroke patients presenting with kidney disease and poor GCS scores. Additionally, researchers are encouraged to conduct further prospective studies to clarify the temporal and causal relationships between these factors and mortality in hemorrhagic stroke patients.

**Nomenclature**
AHR: adjusted hazard ratio
CT: computed tomography
DALYs: disability adjusted life years
GCS: Glasgow Coma Scale
HFCSUH: Hiwot Fana Comprehensive Specialized University Hospital
HICs: high‐income countries
ICP: intracranial pressure
ICU: intensive care unit
IV‐tPA: intravenous tissue plasminogen activator
JGH: Jugal General Hospital
LMICs: low‐ and middle‐income countries
mRS: modified Rankin score
SSA: sub‐Saharan Africa
TIA: transient ischemic attack
WHO: World Health Organization

## Author Contributions

A.T., B.S., A.G.N., and A.S. were involved in the conception and design, acquisition of data, or analysis and interpretation of data. O.K. contributed to data analysis, writing, and editing the document. D.F. gave valuable ideas for the manuscript and revised it critically for important intellectual content.

## Funding

No funding was received for this manuscript.

## Disclosure

All authors read and approved the final version of the manuscript.

## Ethics Statement

The study was carried out under consideration of the Helsinki Declaration of Medical Research Ethics [52]. This work was approved by the Institutional Health Research Ethical Review Committee of Haramaya University College of Health and Medical Sciences (Ref. No. IHRERC/175/2024). Permission was obtained from the Haramaya University College of Health and Medical Sciences administration. The names of patients were not registered in the checklist, and their unique MRN numbers were locked for confidentiality. The need for written informed consent to participate was waived by the Institutional Health Research Ethical Review Committee of the Haramaya University College of Health and Medical Sciences because of the retrospective nature of the study. Data were accessed from September 1 to October 1, 2024. The authors had no access to information that could identify individual participants during or after data collection. Patient data were anonymized, and data access was restricted during extraction.

## Consent

The authors have nothing to report.

## Conflicts of Interest

The authors declare no conflicts of interest.

## Data Availability

The data that support the findings of this study are available from the corresponding author upon reasonable request.
